# Supplementary material for: A Critical View of the Application of the APEX Software (Aqueous Photochemistry of Environmentally-Occurring Xenobiotics) to Predict Photoreaction Kinetics in Surface Freshwaters
Source: Molecules. 2019 Dec 18;25(1):9. doi: 10.3390/molecules25010009 (PMC7017383; doi:10.3390/molecules25010009)
Supplement: Supplementary file 1 [file molecules-25-00009-s001.zip › 3.2.4_gcc-4.4.0.zip!/3.2.4_gcc-4.4.0/3.2.4_gcc-4.4.0/MSYS/doc/msys/README.rtf]

File:	README
Copyright (C):	2001, 2002, 2006
  Earnie Boyd  <earnie@users.sf.net>
Distribution:	See MSYS_LICENSE
MSYS Revision:	1.0.11

Preface:
Ok, you have installed msys and now you're reading this to understand how to get started.  However, I must first explain some important facts about Msys.  Msys file system bindings (mounts) are automatic and happens as described in table 1.  These automatic file system bindings are not changable by the user.  User defined file system bindings can be created by specifying them in the /etc/fstab directory as explained in table 2.

TABLE 1 - Automatic file system maps:
+ ---------------------------------------------------------------------------- +
| The automatic mounts are relative to where the msys-1.0.dll (DLL) is located |
| such that the following is true:                                             |
|                                                                              |
| / - is the parent directory of the directory containing the DLL              |
| /usr - the parent directory of the directory containing the DLL              |
| /tmp - the value of the TMP environment variable                             |
| /c - C:\                                                                     |
| /d - D:\                                                                     |
| . . .                                                                        |
| /z - Z:\                                                                     |
|                                                                              |
+ ---------------------------------------------------------------------------- +

TABLE 2 - /etc/fstab layout:
+ ---------------------------------------------------------------------------- +
| The record format for the /etc/fstab is current a simple one.  I need to     |
| work on the syntax parsing more so that things like embedded spaces work.    |
| If you wish to map a path with spaces you must use the DOS style name.       |
|                                                                              |
| The record format is as follows:                                             |
| d:/some/foo/path /bar                                                        |
|                                                                              |
| As you can see this is simply the Win32 path mapped to a mount point.  It is |
| unix practice to have the /bar created as an empty directory before it can   |
| mount the mount point.  Msys doesn't force this but it will be to advantage  |
| if you do.  Some programs, e.g. find, and some operations, e.g. tab          |
| operate better if the physical directory is present for those mount points.  |
|                                                                              |
| If you create a /etc/fstab record that maps to an automatic mount point Msys |
| will remove it when it adds the automatic point.  This means that at some    |
| small moment in time your mount point actually exists.  However it doesn't   |
| exist long enough to be of any use to you.                                   |
|                                                                              |
+ ---------------------------------------------------------------------------- +

For any executable not dependent on msys-1.0.dll, MSYS will convert POSIX paths that are used as arguments to Win32 paths.  (Prior to MSYS version 1.0.11, an executable was determined to be msys-1.0.dll if it resided in /bin or /usr/bin directories.)  This means that you now have a POSIX environment that will automagically do the right thing w.r.t. changing the paths passed as arguments.  Arguments beginning with a // are considered to be Win32 style switches and will be passed to the program with // converted to / to allow for the command.com/cmd.exe (Win32) style switch.  An example of a Win32 style switch is `write //p /mydocs/msys-rocks.'  In this example write (a.k.a. WordPad) exists in the c:\winnt\system32 path on my system.  The //p becomes /p which tells write to print the document.  And mydocs/msys-rocks converts to c:\msys\1.0\mydocs\msys-rocks so that write can find it.  


STARTING Msys:
Starting Msys should just be a matter of clicking on the MSYS icon on your desktop or Start menu.  If you have the File Manager window open, you may now click on it and have it start also.  Doing these presents you with a console window within which you may enter commands.

Working with MinGW:
If you already have MinGW installed then simply bind the path to MinGW to the /mingw mount point in the /etc/fstab as described above.  If you don't have MinGW installed already then simply unarchive the MinGW tar.gz file in the /mingw directory.  With versions of MSYS prior to 1.0.11, DO NOT unarchive the MinGW tar.gz file in the / directory.  I find it advantageous to now put MinGW and MSYS binaries in the same /bin directory.  It provides one less confusion with where to install your products.

Working with other products:
I find that the easiest thing for working with other products, such as Microsoft Office, is to create a script pointing to the executable.  An example of a script for Microsoft Word on my system looks like the example in Table 3.  I have this stored in my /usr/local/bin directory with a filename of word.  Now all I need to do to edit a word document is `word /mydoc/proposal.doc' and voila up pops a Microsoft Word window with my document in it.

Table 3 - Script to execute MS Word from the command line:
+ ---------------------------------------------------------------------------- +
+ #!/bin/sh                                                                    +
+ start '/c/Program\ Files/Microsoft\ Office/Office/WINWORD' "$@"                +
+ ---------------------------------------------------------------------------- +

This is also advantageous if you find that your program isn't working with RXVT correctly.  RXVT uses PTY emulation and it is often the case that controlling the stdio of a native program isn't functional.  There is no known cure for this PTY issue and I have stopped looking for one.  The above script method is the way you start the processes that don't flush their buffers or cause I/O blocking on the standard I/O channels.

Other documentation:
Be sure to read the MSYS_ series.  I have tried to document how I'm changing the cygwin code to allow us to use it with MSYS.  These can be found in the /doc/msys directory, or you can find them in the /usr/doc/msys directory.  You will also find HOWTO documentation at http://www.mingw.org/wiki/MinGWiki/ so please read it.

Using binaries with different runtimes:
You can't use Cygwin binaries at all and if you try you'll most likely just cause the processes to "hang".  You can use Win32 native binaries but you should put them into the /mingw/bin or your /usr/local/bin directory tree.  If you wish to replace an MSYS binary with a native win32 version then delete or rename the /bin version.


Effective use of the clipboard:
You may use the clipboard with MSYS.  When using rxvt as the terminal, just selecting with data with click and drag of the mouse, copies the data to the clipboard.  To paste the clipboard data in the rxvt terminal you can Shift and Left Click or press both mouse buttons if 3 button mouse emulation is on or press the mouse wheel.  To copy the highlighted data to your favorite windows email client the you use the paste options for that program, typically Ctrl-V.  You can also use interesting bash shell commands such as `cat /dev/clipboard > /tmp/foo' or `less -f /dev/clipboard'.  Data entered into the clipboard by non-MSYS programs can also be used by MSYS programs and vice versa.

Bug Reports:
See http://www.mingw.org/bugs.shtml

User Posts:
MinGW-msys@lists.sf.net

Disclaimer:
Products mentioned in this and other documents are solely owned by their trademark owners.  We claim no rights to those trademarks and any mention of those products are for example only.  Your uses of those products are your responsibility and no endorsement of any mentioned product is being given.
